# Endotypes of occupational asthma

**DOI:** 10.1097/ACI.0000000000000969

**Published:** 2024-01-30

**Authors:** Paola Mason, Marco Biasioli, Filippo Liviero

**Affiliations:** Department of Cardiac-Thoracic-Vascular Sciences and Public Health, University of Padova, Padova, Italy

**Keywords:** biomarkers, irritant-induced asthma, micro-RNA, occupational asthma

## Abstract

**Purpose of review:**

To describe recent findings in endotyping occupational asthma by addressing the role of specific biomarkers.

**Recent findings:**

Studies on occupational asthma endotypes have focused on immune and inflammatory patterns associated with different occupational exposures to sensitizers or irritants.

Sputum neutrophilia has been found in 58.5% patients with occupational asthma caused by high molecular weight (HMW) agents, and work-related dysphonia in patients with occupational asthma was described as associated with sputum neutrophilia too. Neutrophils have been associated also with irritant-induced asthma. The measurement of specific IgE has been confirmed as a valuable diagnostic tool in occupational asthma caused by HMW agents, on the contrary, for most low-molecular-weight agents, the presence of specific IgE has been proven in a small subset of affected workers. Fractional exhaled nitric oxide has been confirmed as a marker of type 2 (T2) inflammation in occupational asthma, mostly when induced by HMW agents (e.g. flour), and it has proved to be more sensitive than spirometry in measuring the efficacy of an intervention.

MicroRNA-155 has been shown to contribute to airway inflammation in occupational asthma induced by toluene diisocyanate.

**Summary:**

Occupational asthma is heterogeneous, thus monitoring multiple biomarkers is crucial to understand, which inflammatory responses are prevalent.

## INTRODUCTION

In 2008, Anderson [1] termed ‘Endotype’ a subtype of disease defined functionally and pathologically by a molecular mechanism or by treatment response. Importantly, nowadays there is a consensus that to achieve the status of endotype, a specific molecular pathway should be identified as critical, if not essential, to the manifestation of that asthma phenotype.

Occupational asthma is a type of asthma in which the inciting agent is encountered in the workplace and can be a high-molecular-weight – HMW – agent or a low-molecular-weight – LMW – agent. Occupational asthma comprises sensitizer-induced and irritant-induced phenotypes.

As described in common asthma, the different phenotypes of occupational asthma are also the consequence of type 2 (T2), non-T2 and mixed inflammation, and across years, many attempts have been made to reveal their complex underlying pathophysiological mechanisms.

To study possible causal endotypes, multiple biomarkers have been studied, mostly represented by eosinophils, neutrophils, total or specific IgE, and fractional exhaled nitric oxide (FeNO). They can be collected at different timepoints, with different sampling methods, and this implicates a variety both of advantages and limitations.

More recently, the attention has moved towards the role of microRNA (MiRNAs) profiles because of their relevance in the pathophysiology of allergic diseases and their potential as biomarkers in liquid biopsies [2^▪^,^3^].

This review deals with recent findings and new challenges in studying occupational asthma endotypes. 

**Box 1:**
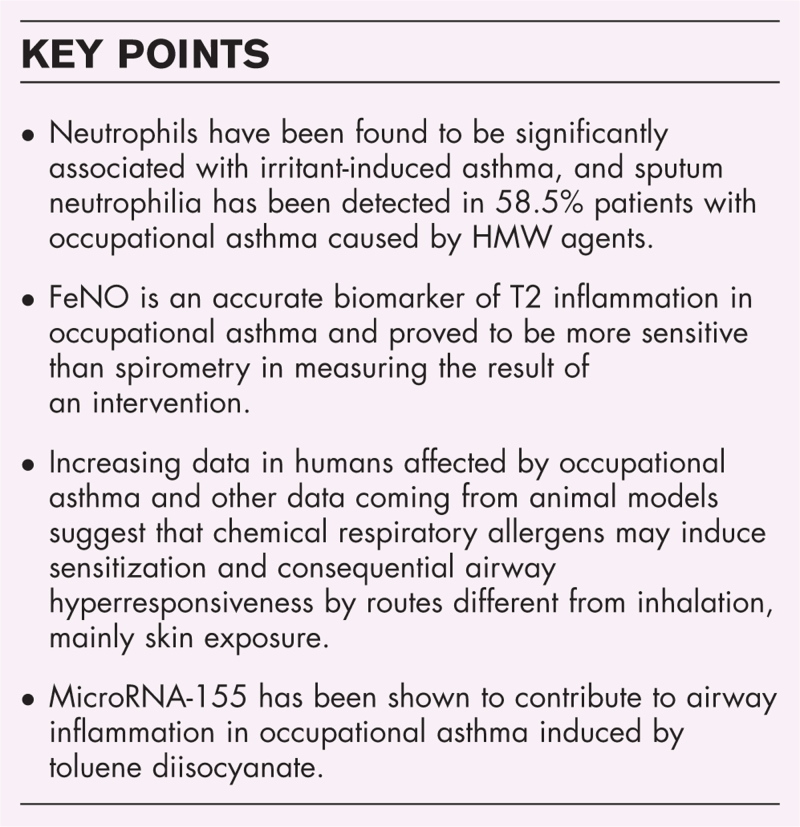
no caption available

## EOSINOPHILIC AND NEUTROPHILIC INFLAMMATION IN OCCUPATIONAL ASTHMA

Quantitative sputum cytometry provides a relatively noninvasive method to assess the cellular component of airway inflammation. The method of sputum collection, processing and the quantification of cell counts are well standardized, and this type of analysis has been used for years in studying occupational asthma [4,5].

According to the airway inflammatory profile, four different patterns are described: eosinophilic (sputum eosinophilia with low neutrophilia), neutrophilic (sputum neutrophilia with low eosinophilia, mixed (high-sputum eosinophilia and neutrophilia) and paucigranulocytic (low-sputum neutrophilia and eosinophilia).

Migueres *et al.*[6^▪▪^] in a multicentre, retrospective cross-sectional study described a cohort of 296 patients with occupational asthma confirmed by specific inhalation challenge (SIC). One hundred and eight were diagnosed with LMW agent occupational asthma and 188 with HMW agents occupational asthma. Induced sputum was assessed before and 24 h after SIC, and ‘Sputum eosinophilia’ was defined by a sputum eosinophil count at least 3% whereas a sputum neutrophil count at least 76% was considered as reflecting ‘Sputum neutrophilia’.

Sputum eosinophilia was associated with high dose of inhaled corticosteroid treatment (ICS), short-acting β2-agonist use less than 1/day while at work, moderate-to-severe level of baseline nonspecific bronchial hyperresponsiveness.

Even though HMW agents were more frequently involved in subjects who showed an eosinophilic pattern, exposure to an HMW agent was not retained as a significant determinant for sputum eosinophilia in the multivariate analysis, thus challenging the traditional concept of categorizing the agents causing occupational asthma into HMW and LMW agents, presuming implicitly that they act through different underlying pathophysiological mechanisms.

Sputum neutrophilia was associated with older age, male gender, short-acting β2- agonist use greater than 1/day, at least two severe exacerbations during the last 12 months at work and isolated early reactions during the SIC.

Notably, a relevant proportion (58.5%) of the subjects with sputum neutrophilia in this cohort were tested with HMW protein agents, mainly flour, while IgE-mediated sensitization to these HMW agents was documented in the substantial majority (83.3%) of these subjects.

These data suggest that an HMW agent can initiate either T2, non-T2, or mixed immune responses. Whether the elicitation of T2 vs. non-T2 immune responses is driven by environmental factors (e.g. endotoxins, multiple chemicals at workplace) and/or host-related (e.g. genetics, airway microbiome) that interact with HMW occupational agents remains to be revealed.

Neutrophil inflammation has been shown to regulate sensory neuron function [7]. Dysphonia is a main symptom of work-associated irritable larynx syndrome, which has been defined as neuronal sensitization by a workplace trigger bringing about laryngeal dysfunction. Hoarseness at work was described in 14.4% of patients with occupational asthma described in the European network for the PHenotyping of OCcupational Asthma (E-PHOCAS) cohort, and when their characteristics were analysed, it was showed that female gender and a higher sputum neutrophil count were significantly associated with a higher likelihood of work-related dysphonia [8].

In dental health workers with mixed exposures to HMW (e.g. latex) and LMW (e.g. acrylates) agents, Singh *et al.*[9] aimed to group occupational asthma subjects based on common inflammatory features and to determine the relationship between these identified features and asthma-associated clinical indices. A group of 76 dental healthcare workers were evaluated. Sera were analysed for atopic status, latex sensitization, and 12 cytokines considered representative of T2-high and T2-low profiles. The classification of asthma forms, based on cytokine patterns, demonstrated both eosinophilic and neutrophilic inflammatory responses. Four phenotypically distinct subgroups relating to the severity of inflammation (acute or chronic) of the cell types were identified. Cytokine determinants for the neutrophilic pattern included IL-1β, 6, 8, 10, 12p70, and TNF-α, whereas for the eosinophilic pattern, these included IL-3, 4, 5, 7, eotaxin, and GM-CSF. The multivariate models documented a significant association between work-related chest symptoms and all four inflammatory patterns. However, this association was stronger for the acute neutrophilic compared with acute and chronic eosinophilic responses, respectively. In this study, neutrophilic phenotypes coexisted with eosinophilic inflammatory phenotypes, suggesting a possible dual pathway for asthma in dental health workers, probably because of mixed exposures at the workplace.

Little is known about pathologic changes in the human airways after exposure to inhaled irritants [10^▪▪^]. Andrianjafimasy *et al.*[11^▪▪^] tried to explore the possible endotypes of irritant-induced asthma by merging data on clinical characteristics and biomarkers of oxidative stress and inflammation. In 999 patients, derived from the EGEA study, they found that occupational exposure to irritants was associated with a distinct respiratory endotype characterized by increased fluorescent oxidation products (a biomarker of damages related to oxidative stress) and highest neutrophil count in blood, thus suggesting oxidative stress and neutrophilic inflammation as potential pathobiological crucial traits.

## IgE ANTIBODIES: BETWEEN CERTAINTIES AND CONTROVERSIES

For years, researchers have tried to answer to the question on the role of IgE in occupational asthma, whether it is causative or an associated phenomenon, and, despite the wealth of research, the debate is still ongoing [12].

The HMW agents are proteins of animal, vegetable or microbial origin acting through an immunological IgE-mediated mechanism. The LMW agents include a wide variety of organic and nonorganic products that may act as haptens and in few cases are associated with an IgE mechanism, namely platinum salts and acid anhydrides. For most other LMW agents, the presence of specific IgE (sIgE) has been proven in a small subset of affected workers [13].

Thus, on one hand the diagnostic measurement of sIgE is often a major step in identifying the precise cause(s) of respiratory allergy, and determination of sIgE to HMW allergens is recommended as a valuable diagnostic tool. However, assays for the measurement of sIgE are commercially available for a limited number of occupational antigens only. Thus, there is the need to develop in-house tests for many agents.

On the other hand, there is a wide spectrum in the ability to detect sIgE for LMW allergens ranging from high detection rate in sensitized to acid anhydrides (sensitivity 81%) to low or very low detection rate in sensitized to isocyanates (sensitivity 21%, specificity 94%) and to plicatic acid (sensitivity 9.6%), respectively [12]. LMW allergens are too small to be recognized by the immune system without conjugation to a protein. Some studies also highlighted the difference in sIgE binding according to the conjugate preparation conditions [14,15].

Tsui *et al.*[16] carried out a retrospective analysis to determine the impact of different asthmagens on the characteristics of occupational asthma, with a focus on the occurrence of prior or concomitant skin disorders.

Of 209 cases of occupational asthma, 66 were caused by HMW agents and 143 by LMW agents. Patients with occupational asthma exposed to LMW agents had higher odds of having (had) allergic contact dermatitis compared with patients exposed to HMW agents. Conversely, HMW agents were associated with higher odds of rhinitis symptoms and high total IgE. The mean level of total serum IgE and the prevalence of high total IgE (>114 kU/l) were significantly lower among patients with LMW-OA than among those with HMW-OA.

Interestingly, in this study, 18% of patients with LMW-OA proved to have a positive patch test result to a workplace agent, thus supporting a potential role of patch testing in identifying etiologic agents and reinforcing the plausibility of occupational asthma.

Increasing data in humans affected by occupational asthma [17–19], and other data coming from animal models [20] suggest that chemical respiratory allergens may induce sensitization and consequential selective airway hyperresponsiveness by routes different from inhalation, mainly skin exposure.

The textile sector is spread worldwide, and reactive dyes are used in many different industries but their effects on health are probably underestimated. They act as skin and respiratory sensitizers and have been shown to cause skin sensitization and/or respiratory difficulties in exposed workers.

As reviewed by Muñoz *et al.*[18], positive skin tests and the presence of sIgE to reactive dyes suggest that respiratory symptoms caused by these LMW agents may be IgE-mediated reactions. It has been suggested by Luczynska and Topping [21] that airborne dye molecules may act as haptens and induce IgE-mediated hypersensitivity reactions.

Lux *et al.*[12] reviewed the diagnostic reliability of targeted sIgE tests for the diagnosis of occupational asthma by a metanalysis of 62 studies.

The pooled pairs analysis indicated a sensitivity of 0.74 and specificity of 0.71 for HMW allergens and a sensitivity of 0.28 and specificity of 0.89 for LMW allergens.

The results for LMW allergens indicated poor discriminatory power with generally low sensitivities and high specificities. A positive result was highly indicative of occupational asthma, whereas a negative test could not exclude the condition because of the high incidence of false-negative results.

## FRACTIONAL EXHALED NITRIC OXIDE: IS IT A MAIN PLAYER IN OCCUPATIONAL ASTHMA?

FeNO is a well tolerated and easy measure to conduct also in an occupational setting. Olivieri *et al.*[22] in a cohort of 174 bakers found that those with work-related asthma, with or without occupational allergic sensitization, had higher levels of FeNO than bakers without respiratory burden, a result that is in contrast with what was previously documented [23].

Healthcare workers are exposed to relatively high concentrations of a wide range of chemicals used for cleaning and disinfection mostly in hospital settings, and more so during COVID-19 pandemic [24]. Rhinitis, asthma and contact dermatitis are the most common work-related diseases in various clinical settings.

Mwanga *et al.*[25] studied 699 healthcare workers exposed to cleaning agents in two Southern African tertiary hospitals to determine the prevalence of work-related respiratory and skin symptoms, allergic sensitization and lung function abnormalities in terms of airway obstruction, bronchial hyperresponsiveness and airway inflammation.

The overall prevalence of doctor-diagnosed asthma was 7%. Atopy was present in 43% of health workers, whereas 4% were sensitized to ortho-phthalaldehyde, 2% to natural rubber latex and 1% to chlorhexidine.

They found that overall 23% of health workers had abnormal (>25 ppb) FeNO; 6% having high (>50 ppb) levels. FeNO was positively associated with sensitization to occupational allergens, primarily ortho-phthalaldehyde and natural-rubber latex, suggesting a possible role of these two agents in underlying allergic airway inflammation in these health workers.

Lantto *et al.*[26^▪^] assessed acute and subacute irritant-induced asthma patients diagnosed during 2004–2018. Sixty-nine patients fulfilled the inclusion criteria, and their characteristics were analysed at the time of the diagnosis and 6 months later. The results were compared with those of two subgroups of sensitizer-induced occupational asthma: 69 HMW and 89 LMW agent-induced occupational asthma patients.

At diagnosis, neither lung function parameters nor NSBH differed between the groups, but the FeNO level was lower in the irritant-induced asthma group than in the other groups. At the control appointment scheduled 6–8 months after diagnosis, patients with HMW-induced occupational asthma showed higher predicted forced vital capacity and FeNO than those with irritant-induced asthma.

In a sequel study performed in the same cohort [27], findings suggested that irritant-induced asthma is more frequently associated with uncontrolled asthma than LMW-induced occupational asthma. Furthermore, older age, a low FeNO value, and uncontrolled asthma at the time of the occupational asthma diagnosis acted as indicators of a poor long-term outcome among those with irritant-induced asthma and LMW-induced occupational asthma.

Oţelea *et al.*[28] published a systematic review in which they assessed the value of measuring FeNO related to three types of airborne exposures: allergens, irritants and respiratory particles inhaled during occupational activities. Thirty-nine studies were included. They concluded that for occupational asthma, there is no consensus on the significant value of FeNO for diagnosis, or on the magnitude of change needed after specific inhalation test or occupational exposure at the workplace. There is anyway some consensus (derived from 8 of 13 studies of the systematic review by Oţelea *et al.*[28]) for the optimal time to measure FeNO after exposure, mainly after 24 h, and FeNO proved to be more sensitive than spirometry in measuring the result of an intervention [29,30].

## MICRORNAs: A GLANCE AT THE FUTURE OF OCCUPATIONAL ASTHMA ENDOTYPES?

MiRNAs act as master posttranscriptional regulators that control most cellular processes. As one miRNA can target several miRNAs, often within the same pathway, dysregulated expression of miRNAs may affect specific cellular responses and contribute, or lead, to the development of many diseases, including asthma [2^▪^].

A particular miRNA of interest is miRNA-155, a critical regulator for IL-33 signalling that affects innate lymphoid cell type 2 (ILC2) expression [31].

Blomme *et al.*[32^▪^] used a mouse model of toluene diisocyanate (TDI)-induced asthma to investigate changes in ILCs and T-cell subsets upon isocyanate exposure. The presence of ILC2 was examined in bronchial biopsies of TDI-induced asthmatic patients. In addition, they exposed miRNA-155 knockout and wild-type mice to TDI to investigate whether miR-155 contributes to isocyanate-induced airway inflammation and hyperresponsiveness. They found that TDI-exposed mice had higher numbers of BAL eosinophils, CD4^+^ T cells and ILCs, with a predominant type 2 response, and tended to have airway hyperresponsiveness. They also documented that TDI exposure-induced IL-33 expression in human bronchial epithelial cells and in murine lungs, which was MiRNA-155-dependent in mice. These data demonstrated that ILCs are involved in TDI-induced occupational asthma.

In common asthma, the role of MiRNAs has been investigated both in T2-high [33] and T2-low [34] endotypes thus suggesting that their study might represent a promising tool for the development of relevant biomarkers in occupational asthma too.

## CONCLUSION

Inflammation, structural, and functional abnormalities within the airways are key features of occupational asthma. Although these processes are quite well documented, they vary across the heterogeneous spectrum of occupational asthma.

Moreover, studies on occupational asthma endotypes are mostly characterized by analysing single or few biomarkers, collected at specific timepoints. Monitoring multiple biomarkers along time would be crucial to understand which inflammatory responses are prevalent.

Finally, we should discharge a concept of steady occupational asthma, and consider that variable occupational stimuli occurring over time can alter the immune and inflammatory profile by acting through specific cellular and molecular mechanisms, and thus inducing a complex dynamic overlap of endotypes and phenotypes.

## Acknowledgements

*None.*

### Financial support and sponsorship

*None.*

### Conflicts of interest

*There are no conflicts of interest.*

## REFERENCES AND RECOMMENDED READING

Papers of particular interest, published within the annual period of review, have been highlighted as:

▪ of special interest
▪▪ of outstanding interest
